# Symbiotic  8: Parallel and Targeted Test Generation (Competition Contribution)

**DOI:** 10.1007/978-3-030-71500-7_20

**Published:** 2021-02-24

**Authors:** Marek Chalupa, Jakub Novák, Jan Strejček

**Affiliations:** 8 Universidad Autónoma de Madrid, Madrid, Spain; 9 University of Twente, Enschede, The Netherlands; Masaryk University, Brno, Czech Republic

## Abstract

The setup of Symbiotic  8 for Test-Comp  2021 brings radical changes in the test generation for coverage-branches property. Similarly as in Symbiotic  7, we generate tests by running our fork of symbolic executor Klee on the analyzed program. Symbiotic  8, however, runs several instances of Klee in parallel. We run one instance of Klee on the original program and, simultaneously, we create one (intentionally unsound) program slice for every program-terminating instruction in the program and run Klee on these slices. Apart from this principal change, we also improved other components of the tool, mainly the program slicer. Further, our fork of Klee now supports symbolic pointer arithmetics and comparison of symbolic addresses.

## 1 Test-Generation Approach

Symbiotic [3,2] is an open-source program analysis framework that combines static analyses with code transformations in order to enable faster analysis of the code. In the setup for Test-Comp 2021, Symbiotic uses *program slicing* [6] in combination with *symbolic execution* [5].

Static (backward) program slicing [6] is a technique that removes program instructions that have no influence on reachability or the effect of selected parts of the program. In Test-Comp, we use program slicing for all properties. For `coverage-error-call` property, we slice the program to remove instructions that cannot affect reachability of the error location. For `coverage-branches` property, we use program slicing to create modified versions of the program on which we are likely to quickly generate tests that reach hard-to-cover parts of the program.

Symbolic execution [5] is a program analysis technique that enumerates all possible execution paths of a program. For every path, it computes its corresponding *path condition*, which is a collection of constraints on program inputs that forms the necessary and sufficient condition to follow the path. Each path condition is then used to create a test that makes the program execute the given path.

### 1.1 Workflow of Symbiotic 8

The workflow of Symbiotic 8 in Test-Comp 2021 for the property `coverage-error-call` is the same as in Symbiotic 7: we slice the analyzed program with respect to calls of the error function and run Klee on this sliced program. If Klee finds a feasible path that calls the error function, we attempt to replay this path in the unsliced program to fill in the possibly missing values returned from calls to functions `__VERIFIER_nondet_*` that may have been sliced away.

The workflow for the property `coverage-branches` changed significantly in Symbiotic 8. For this property, we run several instances of Klee in parallel: one instance on the original program and other instances on slices generated for every terminating location in the program.

More precisely, we create a pool of processes that keeps running at most 8 processes at the same time (on the first-come-first-served basis). We start an instance of Klee on the original program and add it to the pool. Then we identify instructions in the program that terminate the execution (further referred to as *targets*). For each target, we create a slice and queue a run of Klee on this slice.

These slices are *unsound* in the sense that they do not preserve all execution paths to the targets. A slice is constructed in two steps:

1. We gather all instructions that are backward-reachable from the target in the target's function and recursively in the callers of the target's function. However, we move only up the call stack and do not submerge into procedures during this process.
2. After we gather all such instructions, we replace all other instructions with a call to `abort` and apply standard program slicing with respect to the target.

For example, consider the code on the left in Figure 1. It contains three possible targets, namely `error()` (line 7), `abort()` (line 13), and `return 0` (line 17). If we slice with respect to the target `error()`, we start searching the program backwards from this target and get all instructions in the body of function `foo`. Then we pop up from the call to line 16 and collect all instructions of function `main` except the call to `abort` (from which the call to `foo` is unreachable). All instructions except the gathered ones are replaced with a call to `abort`. Standard program slicing then produces the program depicted in the middle in Figure 1 (in this case, it just removes the `return`). The slice for the target `abort()` preserves only three first lines of `main` as depicted on the right in Figure 1.

Whenever the *main* instance of Klee finishes tests generation, we have tests for all feasible execution paths of the program. Therefore, we kill all other running instances of Klee and discard tests that were not generated by the main instance to reduce the size of the test suite. If the main instance does not finish before timeout, we keep all generated tests.

Using the unsound slices aims only to help reaching hard-to-cover places in the program. In particular, potentially expensive detours are replaced by `abort` and symbolic execution thus does not waste resources to discover them (see line 2 in the middle in Figure 1). The current construction of unsound slices

```
int inc(int x) {
    return x + 1;
}

void foo(int x) {
    if (x > 0)
        error();
}

int main() {
    int y = nondet();
    if (y < 0)
        abort();
    if (y == 0)
        y = inc(y);
    foo(y);
    return 0;
}
```

```
int inc(int x) {
    abort();
}

void foo(int x) {
    if (x > 0)
        error();
}

int main() {
    int y = nondet();
    if (y < 0)
        abort();
    if (y == 0)
        y = inc(y);
    foo(y);

}
```

```
int main() {
    int y = nondet();
    if (y < 0)
        abort();

}
```

**Fig. 1.** And example of a program (left) and its unsound slice with respect to the call of `error()` (middle) and `abort()` (right).

guarantees that if a test covers a target in the corresponding slice, then it covers the same target also in the original program. The opposite implication does not hold due to the unsoundness. Note that tests generated from the slices may not and usually do not cover all branches in the original program, therefore we still need to run Klee on the original program.

## 2 Software Architecture

All parts of Symbiotic 8 use llvm 10 [7]. We compile the analyzed program into llvm bitcode by the compiler Clang.

To carry out symbolic execution, we use our fork of the open-source symbolic executor Klee [1]. The fork has several modifications compared to the mainstream Klee. The main modification is the representation of pointers as segment-offset pairs that enables symbolic-sized allocations. Since this year, our fork Klee also supports comparison of and arithmetic on symbolic pointers. We use Z3 [4] as the SMT solver in Klee. The components of Symbiotic are programmed in C++ and the scripts that schedule and control running these components are written in Python.

## 3 Strengths and Weaknesses

Although symbolic execution is very good in generating test-cases, it suffers from the *path explosion* problem. This problem emerges on programs that contain many branching instructions or loops with the number of iterations dependent on the input and may hinder symbolic execution from exploring "deep" parts of the

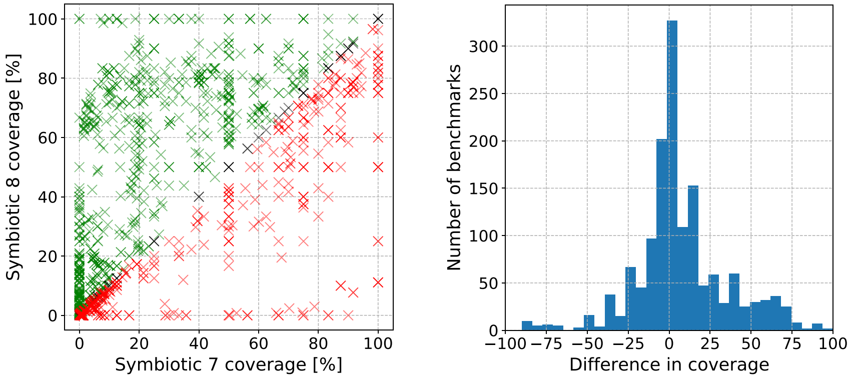

**Fig. 2.** The coverage achieved by SYMBIOTIC 8 and 7 on individual benchmarks of the *Cover-Branches* category program. Using unsound program slices for terminating instructions attempts to alleviate this problem. Although the slice is not guaranteed to preserve paths to the target for which it was created, there are programs where this technique helps symbolic execution to cover substantially more instructions. However, there are also many cases where the technique worsens the coverage alike.

Figure 2 illustrates the overall positive and negative effect of this approach. The scatter plot on the left compares the coverage achieved by SYMBIOTIC 8 and the coverage achieved by SYMBIOTIC 7 on individual benchmarks that were used in both Test-Comp 2020 and 2021.[1] The scatter plot shows that the behavior of the tool changes dramatically. To summarize the data, we compute the difference between the two coverages on each benchmark (for example, if SYMBIOTIC 8 achieves 80% and SYMBIOTIC 7 60% coverage, the difference is +20%). The histogram on the right indicates that the overall effect of unsound slices is positive as the distribution is skewed to positive values. Indeed, SYMBIOTIC 8 won the 3rd place in the category *Cover-Branches* (corresponding to `coverage-branches` property) in Test-Comp 2021 which is a big improvement over the previous Test-Comp, where SYMBIOTIC was 8th out of 9 participants in this category.

The workflow of SYMBIOTIC on `coverage-error-call` did not change from the last year and thus the results are similar.

## 4 Tool Setup and Configuration

The archive is available at https://doi.org/10.5281/zenodo.4491729. Run SYMBIOTIC with the following command

[1] The use of unsound slices is not the only difference between SYMBIOTIC 8 and 7, but we believe that it has the biggest impact on the presented results.

```
bin/symbiotic --test-comp --prp <prpfile> [--32] <source>
```

where `--prp` sets the verified property and `--32` tells Symbiotic to assume 32-bit architecture (64-bit architecture is assumed by default). The generated test-cases are stored in the directory `test-suite`.

## 5 Software Project and Contributors

Symbiotic 8 as it competes in Test-Comp 2021 has been developed by Marek Chalupa and Jakub Novák under the supervision of Jan Strejček. The tool and its components are available under MIT License. llvm, Klee, and Z3 are also available under open-source licenses. The project web page is:

https://github.com/staticafi/symbiotic

## References

[1] C. Cadar, D. Dunbar, and D. Engler. KLEE: Unassisted and automatic generation of high-coverage tests for complex systems programs. In *OSDI*, pages 209–224. USENIX Association, 2008. http://www.usenix.org/events/osdi08/tech/full_papers/cadar/cadar.pdf.

[2] M. Chalupa, T. Jašek, L. Tomovič, M. Hruška, V. Šoková, P. Ayaziová, J. Strejček, and T. Vojnar. Symbiotic 7: Integration of predator and more (competition contribution). In *TACAS*, volume 12079 of *LNCS*, pages 413–417. Springer, 2020. 10.1007/978-3-030-45237-7_31

[3] M. Chalupa, M. Vitovská, T. Jašek, M. Šimáček, and J. Strejček. Symbiotic 6: generating test cases by slicing and symbolic execution. *International Journal on Software Tools for Technology Transfer*, 2020. 10.1007/s10009-020-00573-0.

[4] L. de Moura and N. Bjørner. Z3: an efficient SMT solver. In *TACAS*, volume 4963 of *LNCS*, pages 337–340. Springer, 2008. 10.1007/978-3-540-78800-3_24.

[5] J. C. King. Symbolic execution and program testing. *Communications of ACM*, 19(7):385–394, 1976. 10.1145/360248.360252.

[6] Mark Weiser. Program slicing. *IEEE Transactions on Software Engineering*, 10(4):352–357, 1984. 10.1109/TSE.1984.5010248.

[7] LLVM. http://llvm.org/.

